# Cigarette smoke exposure impairs reverse cholesterol transport which can be minimized by treatment of hydrogen-saturated saline

**DOI:** 10.1186/s12944-015-0160-9

**Published:** 2015-12-03

**Authors:** Chuanlong Zong, Guohua Song, Shutong Yao, Shoudong Guo, Yang Yu, Nana Yang, Zheng Guo, Shucun Qin

**Affiliations:** Key Laboratory of Atherosclerosis in Universities of Shandong Province, Institute of Atherosclerosis, Taishan Medical University, 2# Yingsheng East Road, Taian, Shandong 271000 PR China; School of Basic Medicine Sciences, Taishan Medical University, 2# Yingsheng East Road, Taian, Shandong 271000 PR China

**Keywords:** Cigarette smoke exposure, Hydrogen, Reverse cholesterol transport, High-density lipoprotein, Human CETP transgenic mice

## Abstract

**Background:**

Cigarette smoke (CS) exposure impaired plasma lipid profiles by modification of apolipoproteins. Hydrogen (H_2_) has been proved effective on reducing oxidative stress or improving HDL functionalities in animal models or metabolic syndrome volunteers. This study was undertaken to explore the effects of CS exposure on reverse cholesterol transport (RCT) and the antioxidative effects of H_2_ treatment against CS exposure in mice transgenic for human cholesteryl ester transfer protein (CETP).

**Methods:**

[^3^H]-cholesterol-laden macrophages were injected intraperitoneally into mice, and the samples of blood, bile, liver, and feces were collected for radioactivity determination to evaluate RCT. [^3^H]-cholesterol-laden macrophages were incubated with HDL isolated from different groups of mice, and the samples of cell medium supernatants were collected for evaluating the HDL functionality to elicit cholesterol efflux.

**Results:**

CS exposure significantly decreased plasma HDL cholesterol level (HDL-C) by 22 % and increased LDL cholesterol level (LDL-C) by 21 % compared with the control group (*p* < 0.05, *p* < 0.01), while H_2_ treatment significantly improved the CS-impaired levels of TC, LDL-C and HDL-C by 10, 27 and 31 %, respectively, compared with the CS group (*p* < 0.05, *p* < 0.01 and *p* < 0.05). Besides, CS exposure significantly decreased [^3^H] tracer concentrations in liver, bile and feces by 17, 35 and 48 %, respectively, compared with the control group (*p* < 0.05 for liver and feces), while H_2_ treatment significantly improved them by 21, 72 % and 89 %, respectively, compared with the CS group (all *p* < 0.05). Furthermore, CS exposure significantly decreased the HDL functionality to elicit cholesterol efflux by 26 % (*p* < 0.05), while H_2_ treatment also improved it by 32 % (*p* < 0.05). We did not find any significant alterations in protein expressions of RCT involved genes.

**Conclusions:**

These findings provided direct evidence supporting the notion that CS exposure in vivo impairs plasma lipid profiles, HDL functionalities and macrophage-to-feces RCT pathway in CETP transgenic mice, all of which can be minimized by treatment of H_2_-saturated saline.

## Background

Reverse cholesterol transport (RCT) is generally defined as cholesterol efflux from peripheral cells to plasma HDL uptaked by hepatocytes for utilization, direct secretion into bile and feces disposal [1, 2]. Therefore, the intact HDL functionalities have been believed to be atheroprotective in atherosclerosis development [3]. Cholesteryl ester transfer protein (CETP) facilitates removal of cholesterol ester from plasma HDL in exchange for triglycerides from low-density lipoproteins or very low-density lipoproteins (LDL or VLDL) [4, 5]. Since wild C57BL/6 J mouse lacks CETP gene necessary for simulating human lipid profiles [6], human CETP transgenic heterozygous mice with a C57BL/6J background were used in the present study.

Both animal and human studies have demonstrated that cigarette smoke (CS) exposure significantly increased levels of plasma total cholesterol (TC), total triglyceride (TG), LDL-cholesterol (LDL-C), but decreased the level of HDL-Cholesterol (HDL-C) [7–9]. A few in vitro studies revealed that CS exposure promoted the modification of lysine amino residue on HDL which would thus lose the functionality to stimulate cholesterol efflux from macrophages [10, 11]. On the other hand, our previous studies demonstrated that H_2_-rich water or H_2_-saturated saline decreased aortic atherosclerosis, improved HDL functionalities, and reduced the oxidative stress in animal models or metabolic syndrome volunteers [12–14]. Based on these findings, we proposed that CS exposure in vivo impairs HDL functionalities or RCT pathway in CETP transgenic mice, and that the impairment by CS exposure could be minimized by treatment of H_2_-saturated saline.

## Methods

### Materials

Acetylated low density lipoprotein particles (ac-LDL) were prepared from 48 mL of fresh blood from a healthy volunteer by sequential ultracentrifugation [15]. [^3^H]-labeled-cholesterol was purchased from PerkinElmer (Waltham, MA, USA). Enzymatic assay kits for plasma lipid determinations were purchased from BioSino (Beijing, China). Kit for measuring formation of thiobarbituric acid reactive substances (TBARS) was from Jiancheng Biochemistry (Najing, China). All antibodies were purchased from Abcam (Cambridge, MA, USA). Commercial Taishan cigarettes (11 mg of tar,1.1 mg of nicotine,12 mg of carbon monoxide, Shandong, China) are used in the present study.

### Animals and grouping

Mice of human CETP transgenic strain with a C57BL/6J background were presented by Dr. Xiancheng Jiang (Department of Anatomy and Cell Biology, SUNY Downstate Medical Center). All CETP transgenic mice were offspring littermates of CETP heterozygous mice. Seventy-two male CETP transgenic mice, 10 weeks old, fed a chow diet (Keaoxieli Co. LTD, Beijing, China) and water ad libitum, were housed in a temperature and humidity controlled room with a 12/12 h light–dark cycle. Mice were exposed to either room air or CS at five cigarettes/d and 5 d/wk for 12 weeks. During the last four weeks the mice were intraperitoneally injected with H_2_-saturated saline or vehicle (saline) once daily. All animal experiments were conducted in accordance with the Guidelines for Care and Use of Laboratory Animals of Taishan Medical University, and this study was approved by the Laboratory Animals’ Ethical Committee of Taishan Medical University.

The animals were randomly divided into the control group, the H_2_ group (H_2_-saturated saline treated, 5 mL/kg/d), the CS group (CS exposed) and the H_2_ + CS group (H_2_-saturated saline treated plus CS exposed) (*n* = 18). In addition, a group of eight C57BL/6J mice were added in the experiment of plasma lipid determination to verify the phenotype of CETP transgenic mice.

### Equipment for CS exposure

Cages for CS exposure were re-designed according to Escolar et al. [16]. Briefly, the cages (42.5 × 26.6 × 19 cm) were equipped with a disposable filter cover having 15 10-mm holes that enabled inside air to be flown out of the cages and thus to be continuously renewed. CS was produced by cigarette burning and introduced into the chamber with airflow generated by a 50 mL medical syringe manipulated by a researcher, at a rate of 125 mL/min. Five cigarettes were smoked one by one within 20 min. A rodent ventilator (Ugo Basile, Shanghai, China) was used to provide fresh room air for dilution (1:8) of smoke stream.

### Preparation of H_2_-saturated saline

H_2_ was produced with a self-designed apparatus and immediately dissolved into normal sterile saline to supersaturated concentration for 2 h under high pressure (0.6 MPa). H_2_-saturated saline, stored under atmospheric pressure and 4 °C, was freshly prepared every 3 days to ensure a constant H_2_ concentration of not less than 0.6 mM.

### Preparation of [^3^H]-cholesterol-laden macrophages

Briefly, as described by Yu et al. [17], Raw264.7 cells were incubated with [^3^H]-cholesterol (5 μCi/mL) and ac-LDL (100 μg/mL) for 48 h, and were then harvested at 1.0 × 10^6^ cells/mL for intraperitoneal injection into mice. The ratio of the intracellular [^3^H]-cholesterol radioactivity to the total [^3^H]-cholesterol radioactivity was more than 95 %, which indicated the harvested macrophages had already uptaken 95 % of the total [^3^H]-cholesterol and thus were in good condition.

### RCT assay by [^3^H]-labeled-cholesterol tracing

After CS exposure or H_2_-saturated saline treatment, nine mice from each group were injected intraperitoneally with 0.5 mL of [^3^H]-cholesterol-laden macrophage suspension. At 0, 6, 12 and 24 h after injection, blood was collected from the retro-orbital sinus with heparinized capillary tubes without dietary exposure for 12 h. The mice were then anesthetized with aether and sacrificed at 24 h after injection. Samples of bile, liver, and feces were collected, measured or weighed, and stored at −80 °C for radioactivity determination (*n* =9). Plasma samples were isolated from blood by centrifugation for liquid scintillation counting.

### Cholesterol efflux from macrophages

The cholesterol efflux experiments were performed according to Smith et al. [18]. HDL was isolated from each group of mice by sequential ultracentrifugation. Raw264.7 macrophages at 50 % confluence were co-cultured with acLDL (100 μg/mL) containing [^3^H]-cholesterol (1 μCi/mL) in RGGB (RPMI 1640 supplemented with 50 mM glucose, 2 mM glutamine and 0.1 % BSA) for 30 min. The macrophages were washed with PBS containing 0.1 % BSA, equilibrated with RGGB for 24 h, and cultured for 16 h in the presence of 300 μM dibutyryl cyclic AMP (dBcAMP). The culture medium was then replaced with RGGB containing 200 μg/mL of HDL isolated from each group of mice. The medium supernatants were collected for radioactivity determination after 12 h of incubation. The macrophages were washed and dissolved in 0.4 mL of 0.1 M sodium hydroxide, and lysate radioactivity was measured. The percentage of cholesterol efflux could be calculated by dividing the supernatant-derived radioactivity by the sum of the supernatant-derived radioactivity and the intracellular macrophage radioactivity (*n* = 6).

### LDL oxidation determination

In the presence or absence of the HDL (200 μg/mL) isolated from each group of mice, LDL (100 μg/mL) isolated from a volunteer was incubated with freshly prepared copper sulfate (10 μmol/L) at 37 °C for 2 h. The extent of LDL oxidation was evaluated by measuring formation of TBARS (*n* = 6) [19].

### Preparation of mice peritoneal macrophages

Nine mice from each group were sacrificed and intraperitoneally injected each with 5 mL of sterile PBS, massaging gently the abdomen for 5 min. PBS was then drawn out and centrifugated at 1500 rpm for 10 min. The cells were collected and frozen at −80 °C for Western blot.

### Real-time PCR

Cellular RNA was extracted with TRIZOL Reagent (Invitrogen, USA). cDNA was synthesized using Reverse Transcriptase (TianGen Biotech, China). Real-time PCR was performed using the SYBR-green PCR master mix kit (TianGen Biotech, China). The data were analyzed by using Rotor-gene Q software (ver. 1.7). Relative mRNA levels were calculated by the 2^-DDCt^ method (*n* = 6). All primers used in this study were listed in Table 1.

**Table 1:** Primers used for real-time PCR

Gene	Primer	Sequence (5’–3’)
PPARγ	sense	GCAGCTACTGCATGTGATCAAGA
	Anti-sense	GTCAGCGGGTGGGACTTTC
ABCA1	sense	CGTTTCCGGGAAGTGTCCTA
	Anti-sense	GCTAGAGATGACAAGGAGGATGGA
ABCG1	sense	GGGAAGTTGATAAAGGATGT
	Anti-sense	GATTCGGGCTATGTATGG
SR-BI	sense	ATCTGGTGGACAAATGGAA
	Anti-sense	GAAGCGATACGTGGGAAT
apoA-I	sense	GGCACGTATGGCAGCAAGAT
	Anti-sense	CCCAGAAGTCCCGAGTCAAT
ABCG5	sense	AGCGTCAGCAACCGTGTC
	Anti-sense	AGCAGCGTGGTCTTCCCT
ABCG8	sense	TTAAGCCACTCCCAATACA
	Anti-sense	GTTGCTCCAAGAATAAATGA
ABCB11	sense	CAAATAAGGTTGTGGGTAA
	Anti-sense	AGGACTGACAGCGAGAAT
ABCB4	sense	CCCCACAGAGGGTAAGAT
	Anti-sense	CCAACCAGGGTGTCAAAT
LXRα	sense	TTTGAGCAGCGTCCATTC
	Anti-sense	GCAGTCAGTGAGCCTTCG
HMGR	sense	TGTTCACGCTCATAGTCGC
	Anti-sense	CTCCGCTGTGCTGTTCTG
LDLR	sense	GCCCAAGTCGCCATTCTC
	Anti-sense	GCCTGAGGTCCCATCCAA
CYP7A1	sense	TGGGCATCTCAAGCAAACAC
	Anti-sense	TCATTGCTTCAGGGCTCCTG

### Western blot

Tissue proteins were separated by sodium dodecyl sulfate polyacrylamide gel electrophoresis (SDS-PAGE), and the proteins were transferred onto polyvinylidene fluoride (PVDF) membrane. Relative expression levels of SRBI, ABCG5, CYP7A1, LDLR and ABCA1/G1 were calculated with reference to β-actin (*n* = 5 for CYP7A1 and ABCG5, *n* = 3 for others). All protein bands were quantified using Image-Pro Plus software (ver. 6.0).

### Statistical analysis

All results were expressed as mean ± standard deviation (S.D). To compare the means between groups, One-way analysis of variance test (ANOVA) was performed. Probability values less than 0.05 were considered significant.

## Results

### Neither CS exposure nor H_2_ treatment affects mice body weights

After twelve weeks of CS exposure and four weeks of H_2_ treatment, we compared the data of body weight between different groups at week 12, but did not find any remarkable differences between the control and the CS group, or between the CS and the H_2_ + CS group (Table 2).

**Table 2:** Mice body weight comparisons

Groups	Week0	week8	Week12	*P* value
Control	28.1 ± 0.81	29.5 ± 1.01	31.0 ± 1.81	
H_2_	27.5 ± 0.16	28.4 ± 0.37	29.6 ± 0.81	0.108^*^
CS	28.1 ± 0.47	28.9 ± 1.24	30.1 ± 0.93	0.344^*^
H_2_ + CS	29.1 ± 0.63	29.6 ± 0.61	30.3. ± 1.39	0.819^#^

The *p*-values are calculated from the body weight data between different groups at week12. Values were indicated as means ± S.D. (*n* = 18). *Compared with the control group; #: Compared with the CS group

### CS exposure impairs plasma lipid profiles whereas H_2_ treatment improves

**Fig. 1:**
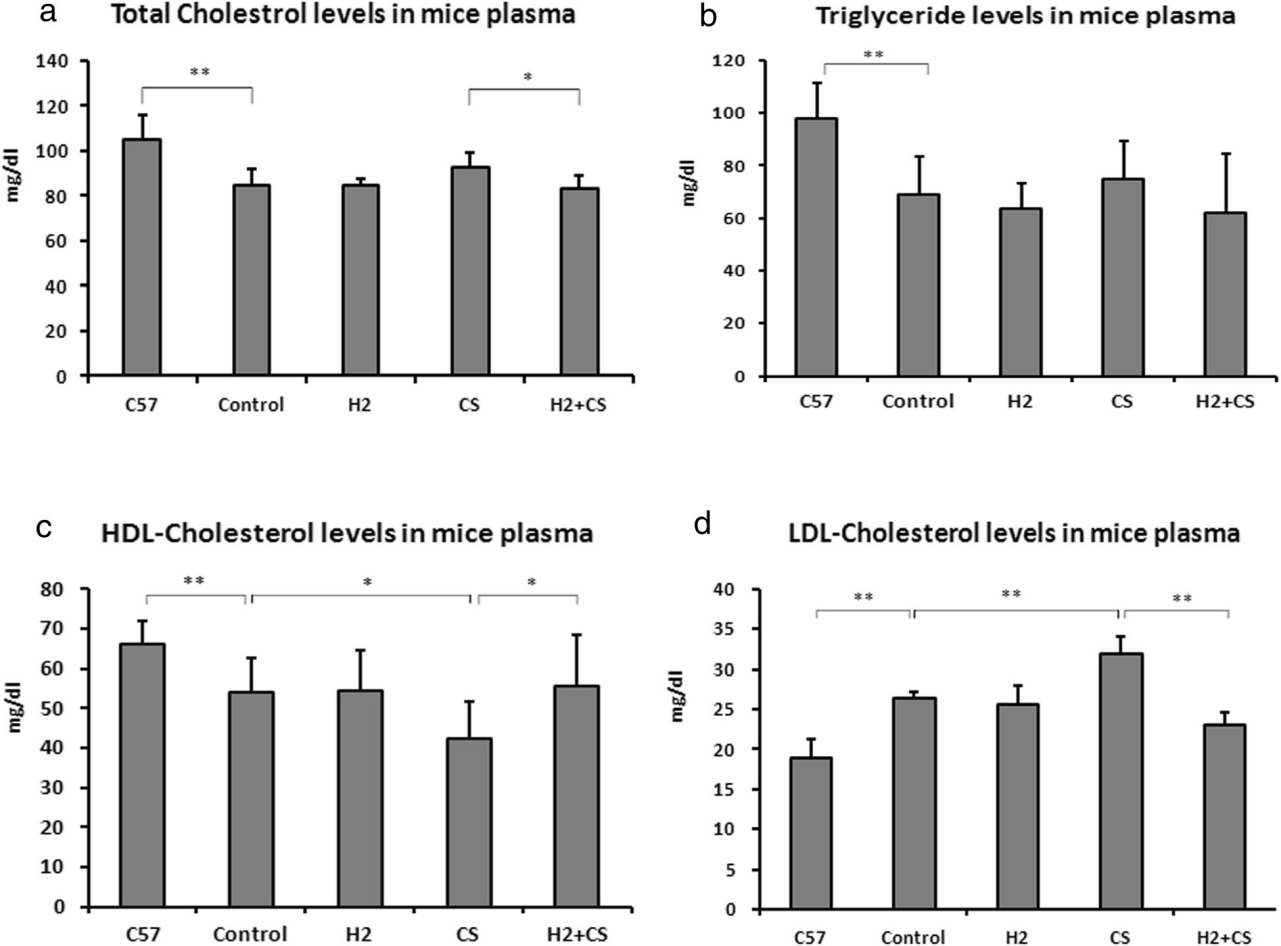
Comparisons between plasma lipid profiles of different groups of mice. The determined lipid profiles included the levels of plasma total cholesterol (Panel **a**), triglyceride (Panel **b**), HDL-cholesterol (Panel **c**) and LDL-cholesterol (Panel **d**). C57 represents the group of eight C57BL/6J mice which were used to testify the phenotype of CETP transgenic mice. Values were expressed as mg per deciliter (means ± S.D, *n* = 8). Significantly different from respective control: *p < 0.05; **p < 0.01

To ensure that model making of CETP transgenic mice was trustworthy, we determined the plasma lipid profiles of a group of the eight C57BL/6 J mice, and found that they were significantly different from those of the control group of CETP transgenic mice (*p* < 0.01). We also found that CS exposure significantly decreased plasma HDL cholesterol level (HDL-C) by 22 % and increased LDL cholesterol level (LDL-C) by 21 % compared with the control group (*p* < 0.05, *p* < 0.01, respectively), while H_2_ treatment significantly improved the CS-impaired levels of TC, LDL-C and HDL-C by 10 %, 27 % and 31 %, respectively, compared with the CS group (*p* < 0.05, *p* < 0.01 and *p* < 0.05, respectively) (Fig. 1).

### CS exposure impairs RCT pathway which can be minimized by H_2_ treatment

**Fig. 2:**
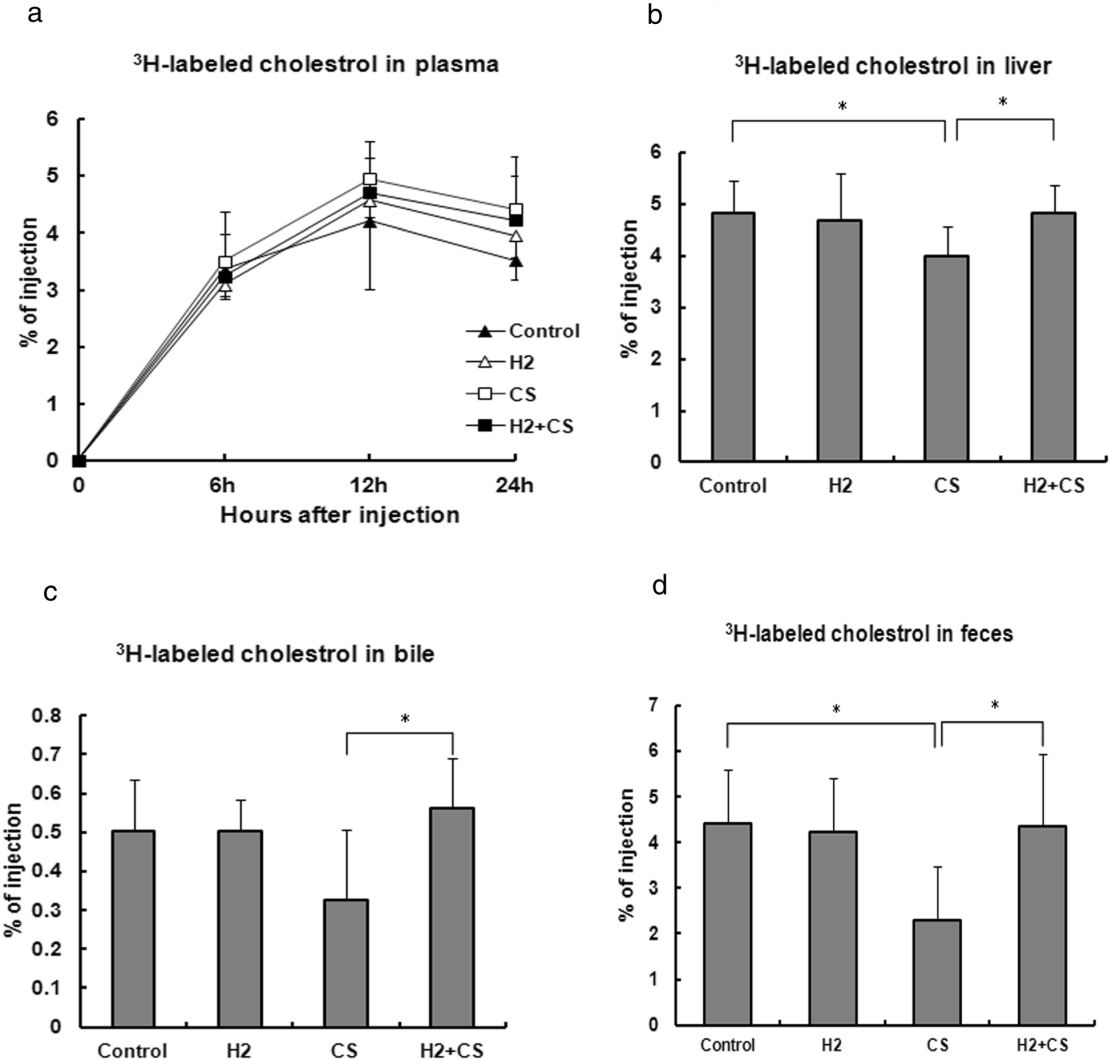
Macrophage-to-feces RCT assay in CETP transgenic mice. Plasma, bile, liver tissue, and feces was isolated or collected for radioactivity determination. At 0, 6,12 and 24 h after injection, blood samples from each group were collected from the retro-orbital sinus for radioactivity determination (Panel **a**). The levels of 3H-labeled cholesterol in liver (Panel **b**), bile (Panel **c**) and feces (Panel **d**) from each group were also determined by liquid scintillation counting. Values were expressed as the percentages of the total injected radioactivity (means ± S.D, *n* = 6 for plasma, *n* = 9 for liver, bile and feces). Significantly different from respective control: *p < 0.05

A classic [^3^H]-labeled-cholesterol tracing assay was employed to evaluate the effects of CS or H_2_ on RCT. As shown in Fig. 2, the plasma radioactivity percentages of the total injection did not differ significantly from each other at each time point (Fig. 2a). Nevertheless, the liver radioactivity percentage of the total injection in the CS group was significantly decreased by 17 % compared with the control group (*p* < 0.05), while the percentage in the H_2_ + CS group was significantly increased by 21 % compared with the CS group (*p* < 0.05) (Fig. 2b). In addition, the bile radioactivity percentage in the CS group was decreased by 35 % compared with the control group, while the percentage in the CS + H_2_ group was significantly increased by 72 % compared with the CS group (*p* < 0.05) (Fig. 2c). Moreover, the fecal radioactivity percentage in the CS group was significantly decreased by 48 % compared with the control group (*p* < 0.05), while the percentage in the CS + H_2_ group was significantly increased by 89 % compared with the CS group (*p* < 0.05) (Fig. 2d).

### CS exposure impairs HDL functionalities which can be minimized by H_2_ treatment

**Fig. 3:**
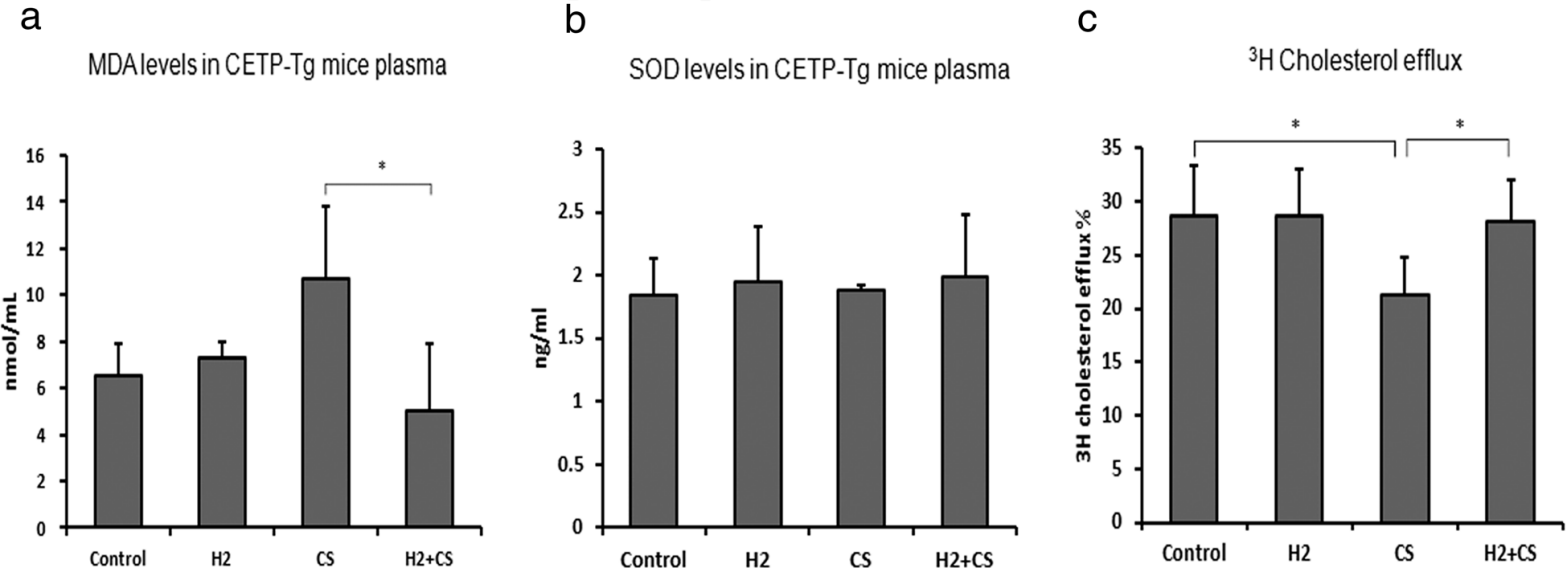
Measurements of HDL functionalities. Levels of MDA, thiobarbituric acid reactive substances (TBARS), were measured to assess the extent of LDL oxidation (Panel **a**). Plasma SOD levels was determined (Panel **b**). [^3^H]-cholesterol-laden macrophages were co-cultured with HDL from each group to determine the HDL functionality to promote cholesterol efflux (Panel **c**). [3H] cholesterol efflux % was calculated by dividing the supernatant-derived radioactivity by the sum of the supernatant-derived radioactivity and the intracellular macrophage radioactivity. Values were indicated as means ± S.D. (*n* = 6). Significantly different from respective control: *p < 0.05

Of all the functionalities, HDL’s protection against LDL oxidation and the functionality to promote cholesterol efflux from peripheral macrophages are very important for RCT pathway. Therefore, we firstly determined plasma TBARS and found that CS exposure significantly increased TBARS formation by 64 % compared with the control group, while H_2_ treatment significantly decreased by 53 % compared with the CS group (Fig. 3a). We also determined plasma superoxide dismutase (SOD) levels by Enzyme-linked immunosorbent assay, but did not find any remarkable alterations (Fig. 3b). When determining the HDL’s functionality to elicit the cholesterol efflux from [^3^H]-cholesterol-laden macrophages in vitro*,* in the presence of cAMP in cell culture medium, we found that CS exposure significantly decreased the efflux by 26 % compared with the control group (*p* < 0.05), while H_2_ treatment also significantly facilitated it by 32 % compared with the CS group (*p* < 0.05) (Fig. 3c).

### Neither H_2_ treatment nor CS exposure significantly alters the expressions of RCT related genes

**Fig. 4:**
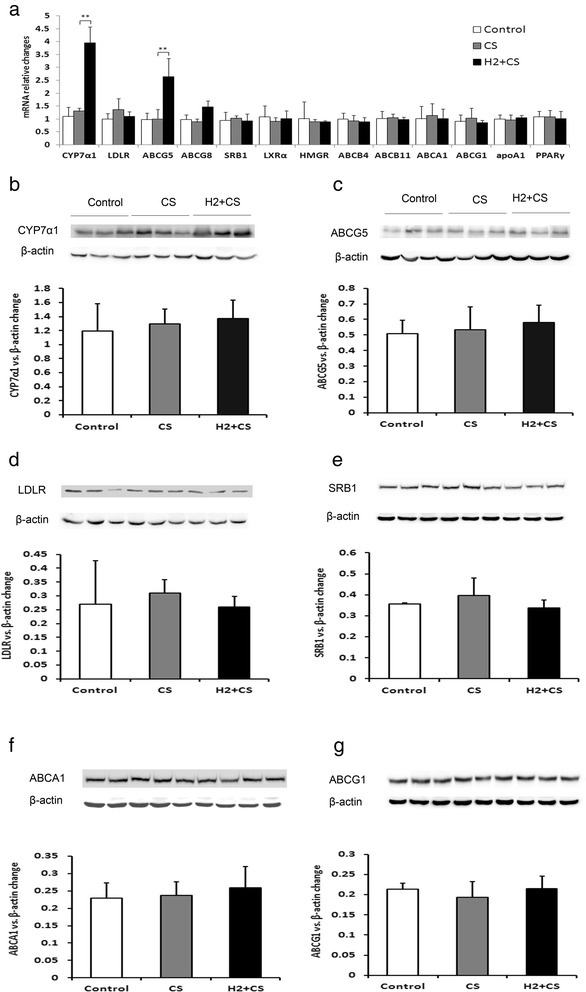
Hepatic expressions of the key cholesterol transporters or enzymes in RCT. mRNA expressions of hepatic cholesterol transporters or enzymes were measured by real-time PCR (*n* = 6) **a**. Values are indicated as relative expression changes compared with respective control. Hepatic protein expressions were measured by Western blots **b**–**e**. Peritoneal macrophages were also measured by Western blot **f**–**g**. Western blot results were shown on upper panels and their quantifications were shown on down panels. Values were indicated as mean ± S.D. (*n* = 5 for CYP7A1 and ABCG5, *n* = 3 for others). Significantly different from the CS group: **p < 0.01

To exclude other possibilities of the underlying molecular mechanisms of H_2_ or CS effect on RCT, the expressions of key cholesterol transporters and cholesterol metabolic enzymes from hepatocytes or peritoneal macrophages were measured by real-time PCR or Western blot. As shown in Fig. 4, although H_2_ + CS treatment significantly up-regulated the mRNA expressions of CYP7A1 and ABCG5 by 294 % and 164 %, respectively, compared with the CS group (Fig. 4a), but neither H_2_ treatment nor CS exposure significantly altered the hepatic protein expressions of CYP7A1, ABCG5, LDLR and SRBI compared with the CS group or the control group (Fig. 4b–e). Besides, when determining the expressions of the cholesterol transporters from peritoneal macrophages, we also found that neither H_2_ treatment nor CS exposure significantly altered the protein expressions of ABCA1 and ABCG1 compared with the CS group or control group by Western blot (Fig. 4f–g).

## Discussion

Cigarette smoking releases a complex mixture of more than 4,700 chemical constituents [20], including aldehydes which are risky constituents for atherosclerosis development. Freeman et al. reported that aldehydes from CS react with lysine residues of lipoproteins, giving rise to adducted molecules in the presence of MDA [10]. Additionally, studies revealed that human and rat cigarette smokers accumulate more macrophages in developing lesions of thoracic and abdominal aortas than non-smokers [21, 22].

Evidence in a variety of biomedical fields has proved that H_2_ acts as a feasible therapeutic strategy in different disease models [23–25]. Ohsawa et al. reported that oral consumption of H_2_ rich water prevented atherosclerosis in apoE-knockout mice fed a chow diet, primarily through decreasing the oxidative stress level in vivo [23, 26]. Our previous studies in animal models and metabolic syndrome volunteers demonstrated that H_2_ supplementation decreased aortic atherosclerosis [12], reduced oxidative stress, improved hyperlipidemia-injured HDL functionality to stimulate macrophage cholesterol efflux [13, 14].

Consistent with previous studies [9, 27, 28], the current study demonstrated that CS exposure could impair the plasma lipid profiles. Interestingly, we also found that H_2_ treatment significantly improved the levels of plasma HDL-C, LDL-C and TC compared with the CS group. These results recommended us that CS exposure or H_2_ treatment might alter the levels of plasma lipids through affecting apolipoprotein structures or enzyme activities on apolipoproteins. Considering that H_2_ helps to reduce oxidation stress [12–14], we proposed that H_2_ prevents certain CS constituents, such as aldehydes, from reacting with apolipoproteins and impairing their functionalities [10]. In other words, before H_2_ treatment CS exposure might impair apolipoproteins on HDL and (V) LDL particles, thus preventing cholesterol uptake from peripheral macrophages to HDL particles and the hepatic cholesterol transfer from (V) LDL particles to hepatocytes via hepatic LDLR or SRBI, leading to a significantly lowered level of HDL-C and increased levels of TC and LDL-C. Whereas, after H_2_ treatment this antioxidant significantly minimized the impairment of plasma lipid profiles, the metabolism of which might be attributable to H_2_ antioxidation for CS-impaired apolipoproteins. Nevertheless, in view of the fact that H_2_ did not have any beneficial effects on non-smokers in this study, H_2_ probably works as a potent antioxidant for oxidative stresses but has little beneficial effect on normal conditions.

The current study also demonstrated that CS exposure reduced the accumulation concentrations of [^3^H]-tracer in liver, gallbladder bile and feces compared with the control group (Fig. 2). In addition, CS exposure not only increased the plasma MDA level but also impaired the functionality of HDL to elicit macrophage cholesterol efflux (Fig. 3). Whereas, nearly all these CS impairments could be minimize by H_2_ treatment. Then, what could be the most possible underlying mechanism for the impaired RCT pathway in the present study? And what is the role of H_2_ throughout the whole impairment of CS?

As is well known, normal plasma HDL helps to decrease the risk of atherosclerotic cardiovascular disease. However, as a result of oxidative stress, HDL particles may undergo dramatic alterations in structure or composition, losing normal biological activities progressively [29, 30]. Such HDL particles cannot protect LDL against oxidation, instead, they turn out to be a kind of dysfunctional HDL and play a proatherogenic role in RCT pathway [30]. McCall et al. also reported that CS exposure could induce a cross-linking of apolipoprotein A1 [31], giving rise to HDL conformational alteration [10, 32]. Besides, the oxidative stress caused by CS exposure has been thought to be associated with plasma antioxidant depletion [33]. It was also reported that volunteer smokers’ plasma MDA levels, a biomarker most frequently used to measure oxidative stress, were enhanced significantly compared with nonsmokers [34]. Therefore, in the present study it was very likely that CS constituents, such as aldehydes, reacted with MDA to form adducts on HDL particles [10], impairing their functionalities and the subsequent RCT pathway. Based on these, it might be concluded that CS constituents impair HDL functionality to stimulate cholesterol efflux and its property to protect against LDL oxidation, and that H_2_ probably helps to prevent MDA to react with lysines of apolipoproteins [10] on HDL particles, thus keeping the HDL functionalities intact upon CS exposure.

Although CS-impaired HDL might uptake less cholesterol from peripheral macrophages, leading to the impairment of lipid profiles, the expressions of key cholesterol transporters and enzymes in hepatocytes or peritoneal macrophages also contribute the lowered or increased levels of plasma HDL-C, TC and LDL-C. To exclude this possibility, we investigated the possible molecular mechanisms for our results (Fig. 4). CYP7A1, the primary rate-limited enzyme in the synthesis of bile acid, is responsible for the main consumption of liver cholesterol. The secretion of bile acid is mediated by ABCG5. LDL Receptors (LDLR) expressed in hepatocytes bind with apolipoprotein E or B, and promote the uptake of cholesterol-containing (V) LDL in hepatocytes. Both ABCA1 or ABCG1 expressed in peripheral macrophages mediates cholesterol efflux to lipid-poor apolipoproteins or mature HDL, respectively. However, as shown in Fig. 4, we did not find that any significant alterations of the expressions of CYP7A1, ABCG5, SRB1, LDLR ABC-A1 or ABC-G1 expressed in hepatocytes or peritoneal macrophages. Thus, we might conclude that the effects of CS exposure on RCT and the antioxidative effects of H_2_ treatment against CS exposure are primarily through impairing or improving the functionalities of HDL, rather than enhancing the protein expressions of cholesterol transporters and cholesterol metabolic enzymes along RCT pathway.

Nevertheless, this study has a list of limitations. Firstly, to further characterize the inner action mechanism of H_2_ on CS-impaired HDL particles, tandem mass spectrometry should be used to elucidate what kinds of peroxide components on HDL particles had been derived from CS impairment and the mechanism of how H_2_ reacts with these derivatives. Secondly, it is well known that lipid-free apoA-I is the lipid acceptor for ABCA1-mediated cholesterol efflux [35–37], while SRBI [38] and ABCG1 [39, 40] are identified as mediators of cholesterol efflux to mature HDL. It remains to be further investigated which apolipproteins or cholesterol transporters should be responsible for the result of cholesterol efflux in this study. Thirdly, a lot of interventions of nutraceuticals have already been investigated on lipid disorders beyond pharmacological interventions [41]. The beneficial actions of nutraceuticals may be related to several mechanisms such as endothelial function amelioration [42], antithrombotic activities [43] and improving lipid metabolism mechanisms [44]. Unlike these nutraceuticals, in view of the fact that the problem for maintenance of H_2_ saturated concentration in cell culture medium has not been addressed, the action mechanism of H_2_ still remains poorly understood. Finally, although one of our previous studies demonstrated that supplementation with H_2_-rich water decreases serum LDL-C, improves dyslipidemia-injured HDL functionalities and reduces the oxidative stress, and concluded that H_2_ might have a beneficial role in prevention of metabolic syndrome [13], further H_2_ atheroprotective studies on humans should still be required to fully understand the mechanism of H_2_ beneficial effects on lipid disorders.

In conclusion, our findings suggested that CS exposure in vivo impairs the plasma lipid profiles, the HDL functionalities and the subsequent macrophage-to-feces RCT pathway in CETP transgenic mice, and that H_2_ treatment decreases plasma MDA level, thereby prevents apolipoproteins to react with MDA, and thus minimizes the CS-impaired RCT pathway.

## Abbreviations

ABCA1/G1: ATP-binding cassette A1/G1
ABCG5: ATP-binding cassette G5
CETP: cholesteryl ester transfer protein
CS: cigarette smoke
CYP7A1: cholesterol 7 alpha-hydroxylase
H_2_: hydrogen
HDL: high-density lipoprotein particles
HDL-C and LDL-C: high-density lipoprotein cholesterol and Low-density lipoprotein cholesterol
LDLR: LDL receptor
MDA: Malondialdehyde
RCT: reverse cholesterol transport
SRBI: scavenger receptor class B type I
TBARS: thiobarbituric acid reactive substances
TC and TG: total cholesterol and Total triglyceride
